# How do transgender young people experience talking about trauma with services?

**DOI:** 10.1177/13591045251320673

**Published:** 2025-02-26

**Authors:** Georgia Crockford, Oliver Hawthorne, Tamara Leeuwerik

**Affiliations:** 1 2238 Canterbury Christ Church University, Tunbridge Wells, UK; 2 9705 Tavistock and Portman Trust, London, UK

**Keywords:** Transgender, gender identity, trauma, trauma-informed care, therapeutic relationship

## Abstract

**Background:**

Transgender young people are more likely than their cisgender peers to experience trauma. Through talking about trauma, services may be able to support transgender young people to manage the impact of these events. However, research has highlighted that many trans people are concerned that disclosing trauma would be used to discredit their sense of their gender identity.

**Aim:**

To explore how transgender young people experience having conversations about trauma with services and how they understand these conversations.

**Method:**

Six semi-structured interviews were carried out with young transgender people. Interpretative phenomenological analysis was used.

**Results:**

The study found that all participants were aware of discourses linking experiences of trauma with transgender identities. All participants recognised these conversations as significant. Some experienced conversations to be supportive and transformative. Others found them deeply distressing, reminiscent of trauma experiences. Relationships with professionals seemed to influence these experiences, as did transphobia and relationships with other services.

**Discussion:**

A trauma-informed approach should be used and professionals are encouraged to consider the potential for harm that can arise from these conversations, as well as the therapeutic element. Clinical implications and future research directions are discussed, in particular considering the recently published Cass Review (2024).

## Plain language summary

Transgender people experience more trauma than the general population. There are different ideas about the relationship between trauma and trans identities. Some think trauma can cause trans identities. Some trans people find this view point harmful and dismissive. Because trans people have high levels of trauma, this study looked at how trans young people found the conversations they've have about trauma with professionals. Six interviews about this topic were held with young trans people who were being seen by the Gender Identity Development Service. This study found that all of these young people had thought about the link between trauma and their trans identity. Some wanted to explore this with professionals. Others were scared of talking to professionals about this. All thought these conversations had had a significant impact on them. For some, talking about their trauma with professionals made a big positive difference to their life. For others, these conversations were upsetting and had features that were similar to trauma experiences. The relationships with the professionals seemed to be important in how people found these conversations. Other factors that affected it included people's previous experiences with services and the way trans people can be treated negatively by wider society. The authors suggest that trauma-informed practices, which are gaining popularity in other areas of clinical practice, should be applied to working with this population. Professionals should be aware these conversations can be harmful as well as positive. Professionals should be are clear about why they are asking about trauma, and what the effects of these conversations might be. This will help people have informed choice about whether or not they want to have these conversations. Professionals should also remember that young people come to services with other experiences that may impact how they find these conversations.

## Introduction

Research is beginning to elucidate that transgender people experience a higher incidence of trauma than the general population. Trans individuals report significantly higher incidence of adverse childhood experiences compared to the general population (Craig et al., 2020; Feil et al., 2023). Given this higher incidence, it is of concern that a recent review of evidence (Cass Review, 2024) found that many young trans people were anxious about discussing this with services due to a worry this would “discredit their sense of identity” (pp. 152) (i.e. they feared that disclosing trauma may lead professionals to conclude their gender identity is a symptom of a mental health problem and that this perception would mean their identity was not viewed as being legitimate). Furthermore, in some situations, fear of discrimination, or treatment being withheld, led some to omit information about trauma and not seek professional support (Ellis et al., 2015).

The topic of trauma and transgender identity has led to intensely polarized debate. Many argue that discrimination due to holding a minoritized identity may account for the higher rates of trauma amongst transgender people, for example Shipherd et al. (2011) found that 98% of a transgender sample (*n* = 97) reported at least one potentially traumatic event, with 42% of people attributing at least one event to bias against their gender identity. Government statistics indicate that hate crimes against transgender people are rising (Hate Crime, England and Wales, 2021). Some researchers argue that experience of trauma may contribute to gender dysphoria. An anonymous, online survey of people who had detransitioned (i.e. stopped identifying as a different gender than that assigned at birth) (Littman, 2021), found that 38% of participants believed that trauma or mental health conditions had originally caused them to experience gender dysphoria. However, it is important to note that this study recruited people largely via snowball sampling of detransition related social media and as such may represent only the views of a subset of those who have detransitioned. Some clinicians with experience working with gender diverse young people also hold this view, for example Evans and Evans (2021). Whilst recently, there have been attempts to think about the relationship between trauma and gender identity from a stance explicitly supportive of transgender identities (Saketopoulou & Pellegrini, 2023), in general this viewpoint has been associated with critiques of social and medical transition. There is strong opposition to this viewpoint with some academics who identify as transgender arguing that this position is harmful and invalidating of trans identities (Ashley, 2019; Horton, 2022). Public, often polarized, debates (e.g. around the relationship between a transgender identity and experiences of trauma) have been described as “dehumanizing” (pp.186) and “invasive” (pp.187) by some trans people (Robertson et al., 2019). The often-polarized viewpoints around the relationship between trauma and gender related distress can also influence professionals, with Canvin et al. (2022) finding some professionals reported feeling inadequately skilled working with this population.

The recent Cass Review (2024) outlines that there is “a need to bring understanding the efficacy of [therapeutic approaches] in line with those used routinely for other children and young people in distress” (pp.31). The review states that thus far, there has been a failure to “systematically consider how psychosocial interventions should be used and research their efficacy” (pp.155). There is an onus on upskilling secondary level services to provide assessment and psychological support for this group, including psychological interventions for trauma. Notably, the Cass Review itself has been met with a polarized response, receiving plaudits (e.g. Abbasi, 2024) and criticism from a number of professional and community groups (see Pearce (2024) for a collation of criticisms). However, the Cass Review will be referred to throughout this paper as it is based on a significant review of available evidence, was commissioned by NHS England and the recommendations are likely to be wide reaching.

Elsewhere in clinical practice, trauma-informed approaches are gaining traction in the UK (e.g. Trauma-informed practice: toolkit; Scottish Government, 2021). Harris and Fallot (2001) highlight the high prevalence of trauma in the general population and stress the need for services to be mindful of this. Trauma-informed care (e.g. SAMHSA, 2014) focuses on several key principles (Table 1). These principles acknowledge the impact that trauma can have on health outcomes and wellbeing, and seek to avoid services retraumatizing users by outlining an approach to interactions between services and users that aims to reduce the negative impact of trauma (see SAMHSA, 2014, p. 11 for definitions of each principle). Whilst there has been research documenting the harm caused by services to transgender people (e.g. Chong et al., 2021; Heng et al., 2018), to date there has been no research exploring specifically how conversations about trauma are experienced by transgender youth.

**Table 1.** Trauma-informed care principles.

Safety	Collaboration and mutuality
Trustworthiness and transparency	Empowerment, voice and choice
Peer support	Cultural, historical and gender issues

Given this background, this study sought to address the following questions:

How do transgender young people experience talking about difficult life experiences in the context of seeking help from professionals around their gender identities?

What sense have these young people made of these conversations?

## Methodology

### Design

Individual, semi-structured interviews with transgender young people were carried out and analysed using Interpretative Phenomenological Analysis (IPA; e.g. Smith et al., 2009; Smith & Nizza, 2021). IPA was chosen as it is concerned with the in-depth exploration of participants’ experiences and their understanding of them. IPA’s idiographic focus allows for capturing shared and unique aspects of experience across the group.

An initial interview schedule was created through reading the relevant literature base, and through one author’s knowledge working in the Gender Identity Development Service (GIDS). Consultation on the topics, questions and procedures was sought from the lived experience stakeholder group at the Gender Identity Development Service. Five responses were received and consultees were compensated with a voucher. The methodology was adapted in response to this feedback. A pilot interview was completed with a trans adult known to the researcher, to increase the likelihood that questions and methods were experienced as acceptable. This resulted in the final interview schedule and procedures.

### Ethical considerations

The study was approved by the Health Research Authority and NHS research ethics committee (Health Research Authority and NHS research ethics committee REC reference: 21/YH/0081). Interviews began with participants verbally reconfirming consent, understanding of confidentiality and their right to withdraw without this affecting care. Participants could choose pseudonyms for quotes. Those who declined had pseudonyms assigned by the researcher. Details of supportive services were provided, and participants identified a supportive person before participating who they could discuss the research with if they felt distressed by the content. All participants had been risk assessed by their clinicians as unlikely to be unduly distressed by participation. Participants were compensated with a £10 shopping voucher; however, they were unaware of this until after the interview to avoid unfair incentivisation to participate.

### Recruitment

Participants were referred by GIDS into the study, in accordance with inclusion criteria (Table 2). GIDS was a national specialist gender service for young people responsible for the assessment and treatment of this population, including psychosocial assessment and endocrinology referrals. Once consent to contact participants had been obtained, further information about the study was provided by the researcher.

**Table 2.** Inclusion criteria.

Aged 14–18
Fluent in English
Current GIDS service-user
Minimum of 3 GIDS sessions attended

### Data collection

Six participants took part (see Table 3 for details). Interviews were conducted online and ranged from 40-90 minutes.

**Table 3.** Participant demographics: As self-identified by participants.

Name	Age	Ethnicity	Gender identity	No. of GIDS appointments attended
**Arlo**	17	White British	Male, transgender	3–4
**Luke**	17	White British	Male	Approx 20
**Becca**	18	White	Transwoman	Approx 40
**Chase**	18	White, Caucasian	Male	15
**Hugo**	17	White British	Male	45–50
**L**	17	White, other^a^	Male	8–10

^a^Ethnicity generalised to ‘Other’ to protect anonymity.

In line with trauma-informed approaches (e.g. Sweeney et al., 2016) which recommend a wide definition of trauma the phrase ‘difficult life experiences’ (DLE) was used instead of ‘trauma’. The term ‘services’ was defined as any setting where participants had encountered professionals, for example school, CAMHS, GPs.

### Data analysis

Data analysis followed IPA procedures (Smith et al., 2009; Smith & Nizza, 2021). Interviews were transcribed verbatim by the researcher, and subsequently read and re-read to develop familiarity with the data. Interviews were analysed on a case-by-case basis. Exploratory notes were made and then developed into experiential statements. From this, personal experiential themes and subthemes were identified for each participant. The whole data set was then considered, and group experiential themes (GETs) were established.

### Reflexivity

Due to the interpretative nature of IPA, researcher reflection on their position to the research is key (Smith et al., 2009). Relevant contexts include the researcher identifying as queer, having lived experience of DLEs and seeking professional support, and working as a mental health professional with experience of working with trans and gender questioning people. To account for the assumptions and biases that might arise from the researcher’s position a bracketing interview (Roulston, 2010) was completed. A research diary was used throughout, and the project was supervised by a GIDS clinician. This aimed to allow for analysis to focus on participants’ experiences, rather than being overly influenced by researcher bias and assumptions. Participants were provided with a summary of findings.

## Results

### Contexts of conversations

Participants had encountered conversations about trauma in a range of services including schools, police, court systems, social services, CAMHS, GPs, gender services, LGBTQ + organisations and therapy. Most participants described a range of positive and negative experiences across settings.

Each interview indicated that discussion of trauma with services had been significant. The analysis generated three overarching GETS and seven subthemes, discussed below (Table 4).

**Table 4.** Group Experiential Themes and Subthemes.

Group experiential themes	Subthemes		
Thinking about the relationship between experiences and gender identity	‘There’s this whole idea’: Awareness of ideas that trauma and transgender identity may be linked	‘I kinda hoped to talk that through’: Wanting to explore the link between trauma and identity	‘I don’t want it to be perceived that way’: Fearing how their experiences would be received
Conversations have a significant impact	**‘**Backed into a corner’: Experiences with services echo trauma experiences	‘You had hardships but you got through it’: feeling better after talking about difficult experiences	‘It depends who you’ve got’: The relationships with professionals and services make a difference
Other life experiences influence young people’s experience of conversations	‘It’s obviously hard to be trans and exist in this world’: transphobia and experiences of other services are important		

### Thinking about the relationship between experiences and gender identity

All participants had heard the idea that trauma may impact on gender identity prior to having conversations about gender with professionals. For each participant, their relationship with this idea significantly affected how they experienced services. For some, awareness of this link inspired curiosity and a desire to explore this with professionals. Others felt concern about how others would respond if they shared their experiences.

#### ‘There’s this whole idea’: Awareness of ideas that trauma and transgender identity may be linked

Participants showed an awareness of societal ideas linking trauma and trans identity.

I saw when I looked at the detransitions a lot of people who were abused as kids that tended to be a reason for transitioning and then detransitioning. So that worried me a bit. (Chase)

[people online] basically say “well all kids who were abused as a kid turn out to be trans, they’re just tryna er deny that that that part of themselves” (Arlo)

#### ‘I kinda hoped to talk that through’: Wanting to explore the link between trauma and identity

For some of the young people, awareness of this narrative made them keen to explore this with services.

Chase expressed “wanting to talk it through”. Others also recognized the importance of this:

For me it’s been something that I needed to talk about (Luke)

It obviously is a significant part and it does need to be talked about (Arlo)

Chase felt that he wanted to talk about these more than some services allowed for.

#### ‘I don’t want it to be perceived that way’: Fearing how their experiences would be received

Several participants recognised that talking about trauma was important but were concerned about how services might respond to this information. Their concerns included sharing trauma impacting their care:

talking about it in that context is scary because you don’t know whether you’re gonna ruin the, your chances of getting the like services. (Arlo)

If you’re not doing well mentally then you won’t get the physical care that you need (Hugo)

Some participants challenged the idea that others might perceive their trauma and their gender as linked and fear it will be used to dismiss them:

It feels like why would that have changed in any way the person who I am? (Arlo)

They’re trying to find something to kind of pick at and be like “mm no I’m gonna discard the rest of this situation” (L)

Some participants also felt that the narrative of trauma and trans identity being linked was used by services to enact a harmful curiosity about trans people and regarded them as a novelty:

maybe I was just a bit interesting to them? Maybe we’re all just a little bit interesting for them? (Hugo)

### Conversations had a significant impact on young people

For all participants, these conversations were significant experiences and had impacted them. Some conversations seemed to share features with trauma experiences, whereas other conversations were experienced as supportive and allowed for personal growth. The relationship built with the professionals seemed to impact how the young person experienced these conversations.

#### ‘Backed into a corner’: Experiences with services echo trauma experiences

Several participants appeared to experience features of these conversations resonating with their traumatic experiences. Hugo named this very explicitly:

It’s traumatic. The like it’s really erm traumatic, like the questions like they ask, the way that they do it, the whole experience has like, yeah, I mean it’s like it’s really traumatic for such young children

Some young people felt they had no choice but to answer questions put to them by services:

There was a lot of discussions which I did not wanna have. (Hugo)

It is part of the whole system that I’ve sort of kind of like accepted that if I’m gonna get treatment I have to just, I have to do it. So. (Arlo)

For some, professional responses to disclosures left them feeling their trust had been damaged:

I felt almost a bit betrayed really because I was like I’ve told you something I haven’t wanted to tell everyone and all of a sudden everyone knows without me being comfortable to tell them. (Luke)

This echoes the damaged trust core to experiences of abuse. Additionally, conversations could feature a power imbalance that some participants felt unable to overcome:

If you’re in a situation where they have semi-authority over you, because they dictate where your referral goes to, you’re kind of backed into a corner and you do sometimes just get forced to answer that question. (L)

Conversations were experienced by some young people as intrusive and invasive, with Hugo directly comparing this to trauma:

The things I was talking about hurt, when they actually happened, they hurt me too much and then the questions that they were asking about it was too intrusive (Hugo)

L also described that once he had answered invasive questions put to him, his experiences were denied by professionals:

Occasionally if I was talking about like trauma they would be like “(tut) you’re a bit young to go through trauma” (L)

In several accounts, participants shared details that can be interpreted as similar to trauma responses. These included difficulty recalling details of the conversations despite strong bodily responses as in Becca’s case:

so I’d I’d sit in meetings and just wait for my nose to bleed and then the clinicians say “ok, that that’s enough for today, you can go”. […] the the weird thing is after the meetings I completely forget about it. I I I I couldn’t tell you what those meetings were about now

Becca also talked about managing these conversations by cutting off from the experience while it happened:

Um… I I remember I I had completely shut down in meetings […] because we're talking about something difficult

This was also highlighted by Hugo, who reported having to disconnect from his emotions while talking to services:

There’s there’s like something in my brain which has like switched off […] because they’ve wanted the answers and I can only give that to them if I switch the feelings off

Strong feelings were evoked for some participants including panic: *“I don’t wanna say anxiety but it’s panic” (Arlo)*, “*pent-up rage” (L),* and feeling used by services:

I well it was like a sense of just feeling quite like like they’d used me. (Hugo)

This left Hugo with a sense of hopelessness that people he turned to for help could make him feel used and *“abandoned”*. L also shared feelings of abandonment and a strong sense of injustice. His understanding was that systemic bias meant that professionals faced no consequences for crossing boundaries:

They’re asking that because they haven’t been told they can't ask that kind of thing. And especially in a system where it's rooted against us, it's like “yeah ask them that, we don’t give a shit”

Hugo and L seemingly hoped they might get support from services after sharing their experiences, however they did not receive this. Instead, they experienced being left alone to manage conversations which made them feel unsettled:

It’s like a soup, you stir up all the soup and then you’ve like you’ve brought it out and it’s all like bubbling and then we’re just gonna leave it (Hugo)

#### ‘You had hardships but you got through it’: Feeling better after talking about difficult experiences

For some, these conversations had powerful positive impacts. Luke states that being able to talk about trauma with services gave him a *“safe space”* to reflect and make room to think about things beyond the trauma:

For them allowing me to let those feelings out kind of uncovered a lot more things that I could talk about, but that I’d never really even thought of so cos the big things were covering them up, like little things that I was struggling with or big things that made me happy which were just covered up. (Luke)

Although Luke found these conversations daunting at first, he regarded them as having *“changed [his] life completely”:*

my confidence levels have gone through the roof like more than I thought they would, I’ve made it to a place I never thought I would be

Similarly, Becca felt these conversations had increased her confidence:

It it made me more confident in myself and less less worried about what people thought of me

Becca and Chase also reflected on what seemed to be a challenging process leading to positive outcomes:

…its…it’s like going into the meetings, they pull you apart so that they can help you put yourself back together[…]and they’re there to help you piece it all together, to help you get a clearer picture of both who you are, who you want to be and who you were and come to terms with the fact that yes, you had hardships but you got through it (Becca)

As hard as it was to talk about, it was a good environment to talk about it[…]it was just like a positive and supportive feedback when I would talk about it. It just sort of helped, it helped. (Chase)

Chase’s experiences in other services resulted in better self-understanding and acceptance, leading to increased coping.

#### ‘It depends who you’ve got’: The relationships with professionals and services make a difference

The relationships with professionals influenced participants experiences of these conversations, with every participant reflecting on experiencing positive and negative relationships.

All participants were able to identify relationships with at least one professional in which they had felt understood. Arlo spoke about an assessment, involving discussion of trauma, in which he felt understood and subsequently calmer:

I was saying “but this wasn’t, this was an isolated incident, this isn’t because of that” and she was like “yeah of course. Like, things have happened at the same time and it’s still significant but it's not a significant part in the development of your er trans identity”[…]I could just breathe

For Arlo, the professional also being trans helped him feel understood. L also had worked with a LGBTQ + professional who he felt understood by, allowing him to explore nuances of his gender and sexual identity.

Some people spoke about the importance of service being aware of the difficulty of these conversations and being attuned to their pacing:

It’s a very big thing, um, and… if- if we did it all at once it would just be so overwhelming um so yeah, it’s it’s good that we just, that we co- that we do it gradually (Becca)

Some participants reflected on the importance of relationships with professionals where it was possible to “*just talk*” (L) about their identity in an atmosphere of non-judgmental acceptance. These conversations may have been particularly meaningful for participants whose families took time to accept their identity.

Obviously family had to kinda come around to it, some faster than others, but [therapist] was the first person to just kinda be like “ok so let's just talk about it” (L)

Having a sense of psychological safety in their interactions with professionals seemed to allow participants the space to express their emotions, and trust that these could be contained:

It it makes me feel supported and it makes me feel like it’s a safe place to um to open up, even if it’s just to just to sit there and have a cry (Becca)

I could just truly say how I felt and why I felt like that and just let my emotions out and like have a cry if I needed to, because I just felt completely safe with the people I was seeing (Luke)

While participants recognized positive relationships, they also reported many instances of professionals distressing them. Sometimes the pacing felt inappropriate. Arlo expressed exasperation at services wanting him to talk about trauma he felt he had moved beyond:

It’s hard to go from a place where I’m at now where I’ve moved on, and I’m proud and I’ve transitioned […] having to go back on that in detail […] honestly it is upsetting

Participants described instances where they had felt misunderstood by professionals when they had shared feelings of distress with them.

He’d be like “yeah well what you’re feeling is just because you don’t get enough sun”. (Arlo)

It was very much just like a “well I’ve gotten my answers you can go fuck yourself, deal with it, you seem fine!” (L)

For some, the feeling of not understanding was reciprocal. When asked about his understanding of why services were asking these questions Hugo responded:

That’s the thing, I wish they– I wish they would’ve told me, cos I don’t know.

Participants also felt unclear around the boundaries of conversations. Some reported sharing something with a professional that was later shared with others and feeling caught off guard by this. They expressed greater need for transparency around boundaries:

It was just the simple thing of saying “oh just to let you know I’m gonna tell your parents” [..] like having that warning instead of one day my parents just coming in and saying “oh by the way, CAMHS told us this” (Luke)

Other life experiences influence young people’s experience of conversations. Further to their current relationships with professionals being important, each participant discussed other interactions with services or wider society that seemed to have impacted how they understood of their conversations with services.

### ‘It’s obviously hard to be trans and exist in this world’: Transphobia and experiences of other services are important

Half of the participants described instances where services had directly intervened in safeguarding them. Experiences of abuse or attacks necessitated a conversation about trauma with a service, e.g. seeking help in school. In one instance, this conversation led to the young person not being allowed to return home.

For Becca and L services had been a direct cause of trauma, with both describing schools discriminating against them. For Becca, this reinforced wider societal messages that trans people are unacceptably different:

I had to use disabled changing rooms for PE. I wasn’t allowed to go into the girls' toilets. I wasn’t allowed to go into the boys' toilets. […] I was an outcast and the school reminded me of that.

Every interview contained reflections on how trans people are viewed in society. Broadly, participants felt trans people are not regarded as mainstream and are unrepresented.

I didn’t know it was possible to be trans, I didn’t know that was a thing (Chase)

Several participants conveyed a sense that trans people were not accepted or understood by others:

It is was really hard […] um, being young and trans. No one understands (Becca)

L experienced this as dehumanising:

We’re not unicorns, we’re not magical beings that you don’t see on a daily basis. We’re people. You know us. We exist. (L)

Four of the young people interviewed reported instances of their LGBTQ + identity as a potential trigger for experiences of being bullied. This was then met with a lack of understanding when they sought help from services:

If I was like being bullied because I was like wearing glasses or something I wouldn’t have to explain that to them. (Hugo)

In addition to direct experiences, participants also heard about services from others (e.g. friends, LGBT spaces). This impacted their perceptions, and perceived sense of safety in services:

We know a lot of other families that’ve gone through this, and erm the vibe is that it’s very much “oh you’ve gotta play the game to get what you want” (Hugo)

You hear the stories of people who have been told “no you’re not allowed to transition, you’re not trans, you’re not valid” (Chase)

Hugo also highlighted that trans joy is overlooked:

The reality is that people think trans people aren’t living happy joyous lives, which just isn’t true. (Hugo)

## Discussion

This study found that all participants were aware of wider societal narratives about the link between trauma and trans identity. Their relationship to this idea was an important frame to their experience of services. Whilst some were keen for support exploring this idea for themselves, others were fearful of the consequences of talking about trauma with professionals.

Whilst all participants felt that the experience of talking about trauma with professionals was significant, their experiences of these conversations varied widely. Some conversations were experienced as negative events, sharing features of trauma. Harris and Fallot (2001), in outlining a trauma-informed approach for systems, highlight themes that characterise abusive relationships. Several of these were present in the participants’ accounts, including betrayal at the hands of a trusted care giver (Luke), the voice of the victim being denied or invalidated (L, Hugo), the victim feeling powerless to alter or leave the relationship (Arlo, Hugo). These experiences of power imbalance and traumatic relational dynamics being repeated within services, seem to stand in contrast with the current move towards trauma-informed practices.

At the same time, some participants reported transformative experiences of talking about trauma. When participants felt safe in therapeutic relationships, they reported that these conversations could “*change* [their] *life completely*”, and increase their confidence and self-understanding. This is in line with wider literature (e.g. Gjerstad et al., 2024) indicating talking about trauma has transformative potential. Other contexts beyond the specific therapeutic relationship also influenced these experiences, including transphobia and previous experiences with services.

### Clinical implications

The Cass Review (2024) makes a number of recommendations about the future of care for this group, including “ultimately skilling up all secondary level services to provide assessment and psychological support for these children and young people” (pp.36). The findings from this study suggest the following should be considered when implementing Dr. Cass’s recommendations:

Trauma-informed principles (e.g. Harris & Fallot, 2001) should be applied to working with this population. Services should be aware of the risk of perpetuating abusive patterns (Harris & Fallot, 2001; Sweeney et al., 2021). Trauma-informed approaches prioritise the levelling of power. Future work with young people should ensure that they have, and are aware of having, true choice about what conversations they enter into, as in trauma-informed approaches with other populations (e.g. Sweeney et al., 2021). People who want to access professional support for trauma experiences should be able to do so. This includes how this relates to their gender identity if they choose to explore this. Attention should be paid to ensure the appropriate setting, timing and relational context for these conversations.

Trauma-informed approaches encourage transparency and the elimination of ambiguity from the outset (Harris & Fallot, 2001). Professionals should be explicit about their aims when asking about trauma. Clarity about the potential outcomes of sharing information with services, for example confidentiality limits and impact on access to medical care, could empower young people to give or withhold informed consent to such conversations. Consideration should be given to how to meaningfully separate these conversations from decision making about medical interventions.

Trauma-informed approaches also should consider the professionals. Trauma-informed supervision, which considers the relationship between the professional, the trauma, the helping relationship and the context in which help is provided (Etherington, 2009) should be delivered to professionals working with this population. This may reduce the risk of vicarious trauma and stress inhibiting professional empathy and potentially resulting in an othering and dehumanising approach to clients.

Another important factor in upskilling this new workforce, is an understanding of the context in which these young people come to services, that is one in which feelings of powerlessness and prior negative experiences leave them fearing invalidating and dehumanizing responses. The polarized political and media context around the lives of transgender people, along with the rising incidence in hate crimes, create a context where young people accessing gender services are likely to experience significant anxiety about who they will meet and how they will be received. Awareness of these contexts and how they impact people’s relationship to help (Reder & Fredman, 1996), if held with thoughtful therapeutic curiosity, may make the positive outcomes reported by some in this study more likely. This could serve to strengthen young people’s relationships with services, potentially improving their overall health outcomes.

### Limitations and directions for future research

The Cass Review (2024) recommends a wide-reaching research programme to inform assessment and therapeutic approaches with this population. To extend trauma-informed thinking to research, a power-levelling approach of collaboration with young trans people in conducting research should be taken. This could expand understanding about how, when and with whom conversations about trauma are held in order to maximise positive outcomes.

It is notable that no non-binary participants or participants from ethnic backgrounds other than white were involved in this study, and only one participant was a trans woman. Considering intersectionality is important as the wider literature shows some evidence that people from ethnic minority groups may disclose trauma at lower rates to professionals than their white counterparts (e.g. Roberts et al., 2011), resulting in increased barriers to seeking support for trauma for those who occupy multiple marginalized identities. Further research should seek to engage and represent previously underrepresented populations. Furthermore, participants were open to GIDS and referred to the study by professionals. This link between the research and gender services may have prevented some young people from engaging with the research for the same reasons as approaching services can be intimidating.

### Conclusion

This study found that young trans people were aware of the discourses surrounding trans identities and trauma, and were significantly impacted by conversations they had with professionals on this topic. Some conversations were supportive and transformative, and it is recommended that researchers and clinicians work with young trans people to further create conditions in which people can safely explore trauma if they choose to. Some conversations were reminiscent of trauma experiences and services should seek to address this in practice by using trauma-informed approaches, ensuring young people are supported to have true choice in entering into these conversations. Clinicians should hold in mind the context of transphobia and negative previous experiences with services that may be impacting this group’s relationship to help.
